# Hypoxia Hits Glucose Metabolism in the Guts

**DOI:** 10.1016/j.jcmgh.2022.01.021

**Published:** 2022-02-12

**Authors:** Cormac T. Taylor

**Affiliations:** School of Medicine, The Conway Institute of Biomolecular & Biomedical Research & Systems Biology Ireland, University College Dublin, Belfield, Dublin, Ireland

Hypoxia, the condition where cellular oxygen demand exceeds supply, is a commonly encountered physiologic stress in the gastrointestinal tract. The intestinal epithelium is particularly susceptible to experiencing physiologic hypoxia because it sits at the interface between the highly vascularized mucosal bed and the anoxic intestinal lumen and is therefore exposed to a steep transmucosal oxygen gradient.^1^ Like virtually all metazoan cells, intestinal epithelial cells have a highly conserved ability to respond to the bioenergetic threat of hypoxia at the cellular level through increasing the expression of genes that promote adaptative processes including angiogenesis and metabolic reprogramming.^2^ The cellular adaptive response to hypoxia is driven primarily (although not exclusively) by the hypoxia-inducible factor (HIF) transcription factor, the identification and elucidation of which resulted in the award of the 2019 Nobel Prize in Medicine or Physiology to Peter Ratcliffe, Gregg Semenza, and William Kaelin.^3^ HIF occurs in 2 main isoforms, termed HIF-1 and HIF-2, which control distinct yet overlapping gene cohorts. The HIF-1 isoform plays an important role in cellular glucose metabolism because it is an important regulator of the glycolytic pathway, whereas the HIF-2 isoform is responsible for the regulation of erythropoietin production in the kidney and liver. In total more than 400 hundred genes are now known to be under the control of either or both HIF isoforms.

The intestinal epithelium is a complex heterogenous monolayer of cells that plays important roles in multiple key processes including innate immunity, fluid homeostasis, metabolism, and enteroendocrine function. The activation of epithelial HIF in response to mucosal hypoxia has been demonstrated to result in the activation of a battery of genes that promote intestinal epithelial barrier function. In fact, pharmacologic activation of the HIF pathway has now been shown in multiple models of intestinal inflammation to provide barrier protection and is currently under investigation in clinical trials for the treatment of ulcerative colitis.^1^ Other studies have demonstrated important roles for HIF in other intestinal epithelial cells including the control of antimicrobial peptide production and mucin secretion from enterocytes.^4^^,^^5^ Therefore, it is now clear that HIF contributes to overall gut health in a highly significant way through the promotion of intestinal epithelial barrier function and innate immunity.

With respect to whole animal glucose metabolism, intestinal L-cells produce GLP-1, a peptide that plays a key role in the control of physiologic glucose homeostasis by increasing pancreatic insulin secretion and reducing hepatic steatosis and adiposity. The understanding of the molecular mechanisms underpinning the regulation of intestinal GLP-1 secretion has been hitherto limited. In the current edition of *Cellular and Molecular Gastroenterology and Hepatology*, Mooli et al^6^ provide convincing evidence that intestinal hypoxia via the HIF-2 isoform drives GLP-1 production from intestinal L-cells through the transcriptional upregulation of the GPR40 and GPR120 lipid sensors, the expression levels of which determines the L-cell’s sensitivity to luminal lipids, such as long-chain fatty acids.^6^

Of interest, the regulation of GLP-1 secretion by L-cells seems to be mostly dependent on the HIF-2 isoform rather than the HIF-1 isoform, which seems to be more important in the regulation of intestinal epithelial barrier function outlined previously. This result underscores the differential effects of these 2 HIF isoforms in the regulation of intestinal epithelial function and the importance of the development of specific HIF-1 and HIF-2 activators.^7^

Although Mooli et al^6^ provide compelling evidence using genetic models for a role for HIF-2 in the regulation of GLP-1 secretion from intestinal L-cells, these data invoke further ideas for the potential therapeutic application of drugs that promote the HIF pathway for the treatment of disease. This is of key importance from a therapeutic perspective because prolyl hydroxylase inhibitors (which activate the HIF pathway) have recently been granted approval for the treatment of anemia in patients. Therefore it is conceivable in the short to medium term that these drugs may be repurposed for the treatment of metabolic diseases, such as diabetes. It will be interesting to see if the results presented by Mooli et al^6^ in the current issue of *Cellular and Molecular Gastroenterology and Hepatology* can be reproduced using pharmacologic means, thereby further opening the window of opportunity for the use of drugs, such as the HIF-activating prolyl hydroxylase inhibitors, for the promotion of glucose metabolism and the treatment of metabolic disease.
